# Mild water stress-induced priming enhance tolerance to *Rosellinia necatrix* in susceptible avocado rootstocks

**DOI:** 10.1186/s12870-019-2016-3

**Published:** 2019-10-29

**Authors:** E. Martínez-Ferri, G. Moreno-Ortega, N. van den Berg, C. Pliego

**Affiliations:** 1 IFAPA. Centro de Málaga. Cortijo de la Cruz s/n, 29140 Churriana, Málaga, Spain; 2 Department of Biochemistry, Genetics and Microbiology, University of Pretoria, Pretoria, South Africa; 3 Forestry and Agricultural Biotechnology Institute (FABI), University of Pretoria, Pretoria, South Africa

**Keywords:** Abiotic and biotic stress, Drought recovery, Fungal pathogens, Gene expression, Priming, Physiological response, White root rot

## Abstract

**Background:**

White root rot (WRR) disease caused by *Rosellinia necatrix* is one of the most important threats affecting avocado orchards in temperate regions. The eradication of WRR is a difficult task and environmentally friendly control methods are needed to lessen its impact. Priming plants with a stressor (biotic or abiotic) can be a strategy to enhance plant defense/tolerance against future stress episodes but, despite the known underlying common mechanisms, few studies use abiotic-priming for improving tolerance to forthcoming biotic-stress and vice versa (‘*cross-factor priming’*). To assess whether *cross-factor priming* can be a potential method for enhancing avocado tolerance to WRR disease, ‘Dusa’ avocado rootstocks, susceptible to *R. necatrix*, were subjected to two levels of water stress (mild-WS and severe-WS) and, after drought-recovery, inoculated with *R. necatrix*. Physiological response and expression of plant defense related genes after drought-priming as well as the disease progression were evaluated.

**Results:**

Water-stressed avocado plants showed lower water potential and stomatal limitations of photosynthesis compared to control plants. In addition, NPQ and *q*N values increased, indicating the activation of energy dissipating mechanisms closely related to the relief of oxidative stress. This response was proportional to the severity of the water stress and was accompanied by the deregulation of pathogen defense-related genes in the roots. After re-watering, leaf photosynthesis and plant water status recovered rapidly in both treatments, but roots of mild-WS primed plants showed a higher number of overexpressed genes related with plant defense than severe-WS primed plants. Disease progression after inoculating primed plants with *R. necatrix* was significantly delayed in mild-WS primed plants.

**Conclusions:**

These findings demonstrate that mild-WS can induce a primed state in the WRR susceptible avocado rootstock ‘Dusa’ and reveal that ‘*cross-factor priming’* with water stress (abiotic stressor) is effective for increasing avocado tolerance against *R. necatrix* (biotic stressor), underpinning that plant responses against biotic and abiotic stress rely on common mechanisms. Potential applications of these results may involve an enhancement of WRR tolerance of current avocado groves and optimization of water use via low frequency deficit irrigation strategies.

**Electronic supplementary material:**

The online version of this article (10.1186/s12870-019-2016-3) contains supplementary material, which is available to authorized users.

## Background

Avocado (*Persea americana* Mill.), a member of the Lauraceae family, is a very important fruit crop consumed worldwide in more than 50 countries. Avocado fruit is considered to be one of the top 15 healthiest foods according to surveys across the United States and Western Europe [1] and is becoming a key component of the consumer’s diet in many countries. Avocado health benefits have triggered its consumption in recent years (~ 4.6% increase of worldwide consumption every year; ~ 25% increase in Europe [2];) but production remains a step behind (~ 4.5% increase per year [3];), which raises concerns about the difficulties of satisfying this demand in the near future.

This gap between production and demand is aggravated by the incidence of avocado diseases, the soilborne pathogen *Phytophthora cinnamomi* Rands (Phytophthora root rot; PRR) being one of the major limiting factors of avocado production worldwide [4]. Given the importance of this pathogen, many studies have been focused on the control of PRR and positive results, derived from an integrated approach involving the use of phosphate, proper field management and commercially available rootstocks with partial tolerance to *P. cinnamomi* (‘Thomas’, ‘Duke 7’ and ‘Dusa’) [5, 6], have been achieved.

Another important soilborne disease affecting avocado groves in productive temperate regions such as South Africa, Israel, Italy and Spain (avocado exporters to the European market), is the white root rot (WRR) caused by *Rosellinia necatrix* Prill [7, 8].. In contrast to *P. cinnamomi*, control of this disease is a complex and difficult task, and, to date, no completely effective control methods have been developed [8, and references therein]. As for *P. cinnamomi*, breeding for *R. necatrix* tolerant rootstocks could represent an effective method for controlling the spread of this pathogen [9] but, although a breeding program is ongoing in Spain (Andalusian Institute of Agricultural Research and Training; IFAPA), no commercial rootstocks are currently available. Thus, alternative approaches, focused on achieving environmentally friendly strategies to decrease WRR incidence in avocado production areas, are necessary.

In this regard, many studies have shown that the pre-exposure of plants to a stress-inducing factor (priming concept) [10–12] allows them to become more tolerant to forthcoming biotic (i.e. pathogens [10, 13]) or abiotic (i.e. water stress, chemical compounds [14, 15]) stress episodes. This priming-induced tolerance seems to be associated with a more rapid and robust activation of cellular defense responses in primed plants compared to non-primed ones [11, 12, 16]. Although the mechanisms underlying the induction of the priming state are complex and diverse [17], it is well known that plant stress responses to biotic or abiotic factors share common pathways [18, 19] and even cross-tolerance can be achieved [20, 21]. For instance, levels of salicylic acid (SA), associated with reactive oxygen species (ROS) signalling and with the regulation of important plant physiological processes [22, 23], have been reported to increase under drought stress [18, 24–26] and pathogen attack [27–30]. More concretely, the accumulation of SA induces the transcription of non-expressor of pathogenesis related gene 1 (NPR1) that further activates genes encoding pathogenesis-related (PR) proteins [31, 32], shown to play an important role in either biotic [33–37] or abiotic stress [38–42] responses. Particularly, avocado tolerance to *P. cinnamomi* and *R. necatrix* has been linked to the induction of PR-genes and protease inhibitors, respectively [19, 43]; both are related with other abiotic stresses such as water stress [44, 45]. Thus, it is possible that exposure to one type of stress (i.e. abiotic stressor) could activate plant responses enabling tolerance to different types of forthcoming stresses (i.e. biotic stressor [46];); hereinafter referred as ‘*cross-factor priming*’. In fact, it has been reported that drought-primed *Eucaliptus* plants were more resistant to *Neofusicoccum* fungal infection compared to non-primed ones [16].

In this context, the present study aims to test whether drought-priming could be used in avocado to increase tolerance to WRR disease. For this purpose, the role of drought-priming in *R. necatrix* interaction with the susceptible avocado ‘Dusa’ rootstock was evaluated by assessing physiological status, stress-related gene expression, and disease progression response.

## Results

### Physiological response of avocado ‘Dusa’ rootstocks to mild and severe water stress levels and recovery after re-watering

**Fig. 1:**
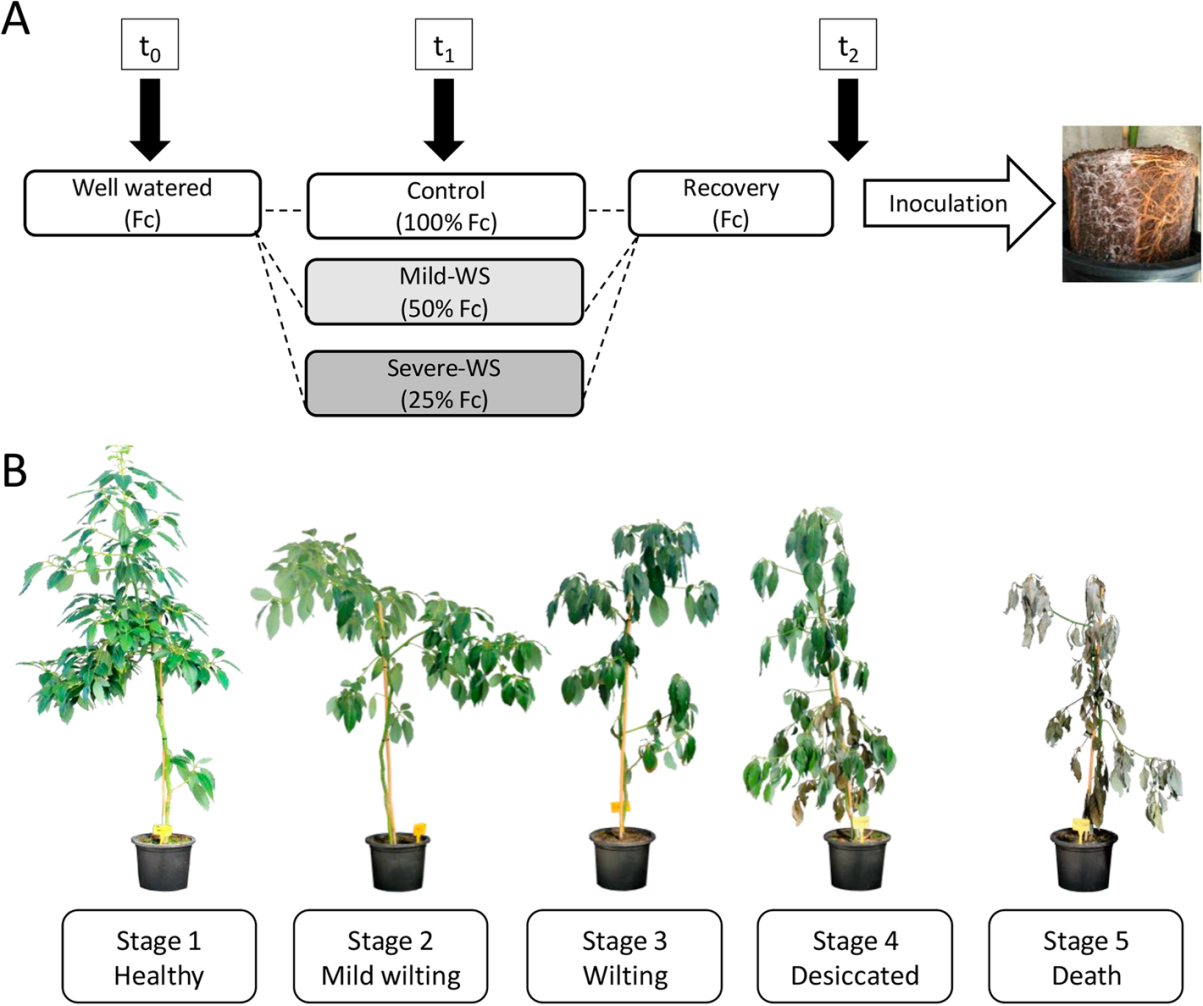
Schematic illustration of the experimental design (**a**) and stages of aerial symptoms in ‘Dusa’ plants inoculated with *R. necatrix* (**b**). Control plants were watered to field capacity (Fc) throughout the experiment and water stressed plants were subjected to controlled substrate drying-up until they reached 50% of Fc (mild-WS) and 25% of Fc (severe-WS), respectively (t_1_). Afterwards, all plants were fully irrigated to assess drought recovery response (t_2_) and to carry out the pathogenicity test with *R. necatrix*

**Fig. 2:**
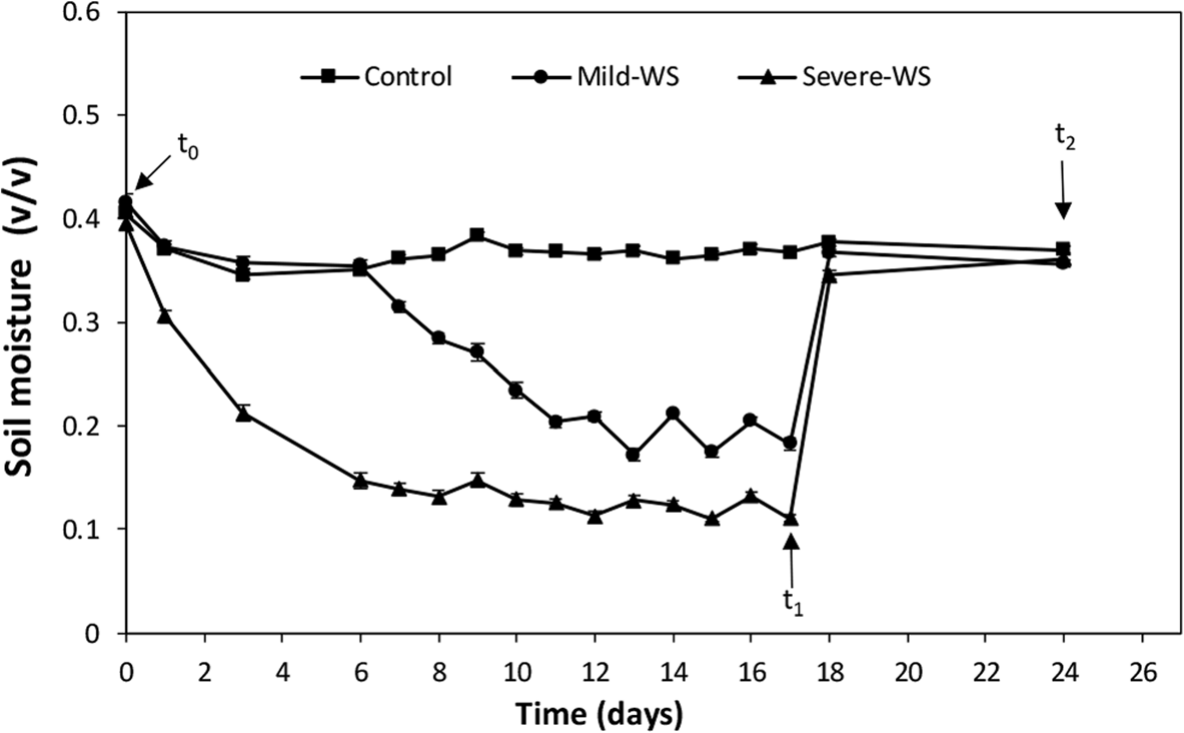
Time-course of mean values (±SE) of volumetric soil moisture of ‘Dusa’ non-stressed control plants (n = 36) and subjected to two water stress (WS) treatments: mild-WS and severe-WS (*n* = 38). The arrows indicate the time points where plants physiological status (t_0_), physiological measurements and root samplings (t_1_, t_2_) were done

To investigate the priming-induced response of ‘Dusa’ rootstock by mild and severe water stress (mild-WS and severe-WS), two sets of well irrigated plants (at field capacity, Fc ~ 0.4 v/v) were subjected to water deprivation until soil water content (SWC) reached 50 and 25% of Fc, respectively (Fig. 1). Throughout the experiment, a set of plants were irrigated daily to act as controls whereas, in the two sets of water-stressed plants, water lessening was done progressively to attain both water stress levels concurrently (after a 6 day lag; Fig. 2). Once these levels were reached, plants were re-watered and Fc values were achieved immediately. Daily irrigation was restored in all plants until inoculation with *R. necatrix*.

**Fig. 3:**
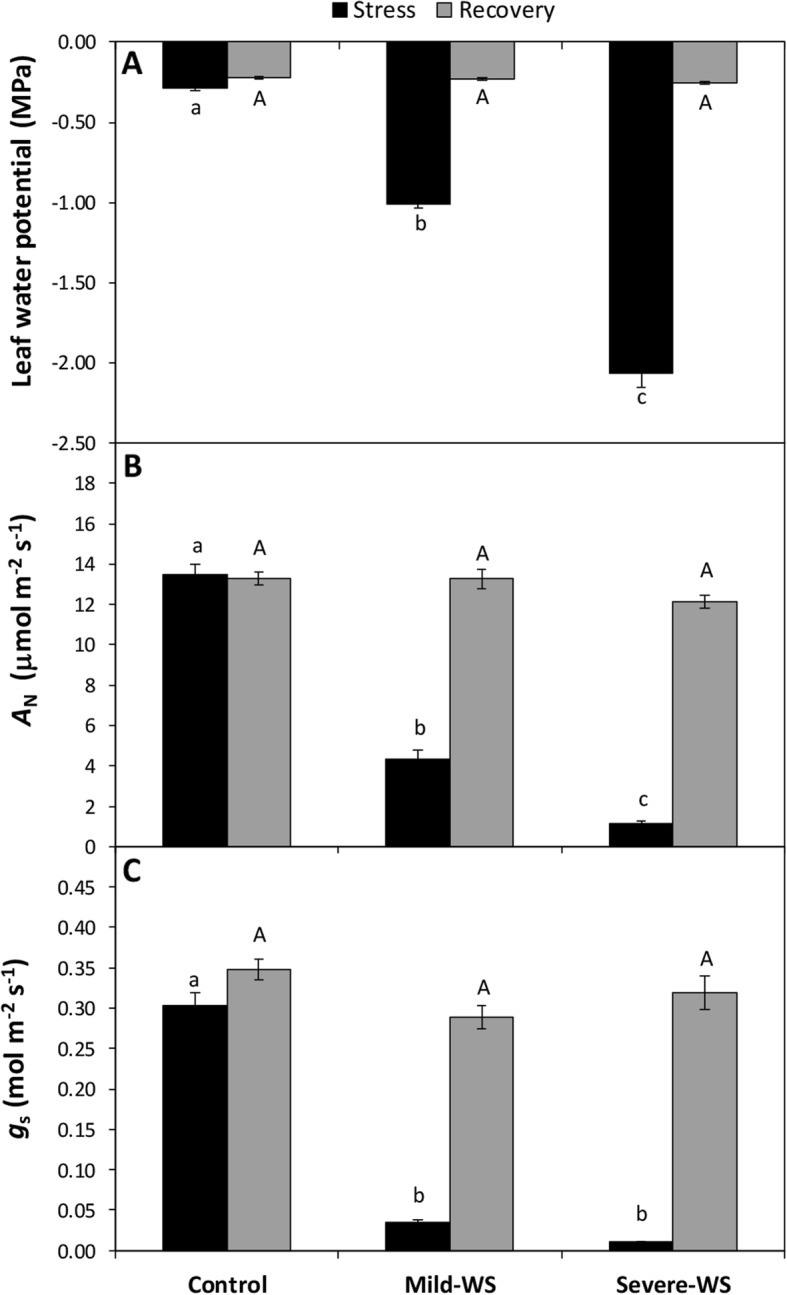
Midday water potential (**a**), net CO_2_ assimilation rate (*A*_N_; **b**) stomatal conductance (*g*_s_; **c**) of ‘Dusa’ plants in control, mild-WS and severe-WS water treatments. Black and grey bars show mean values (±SE, *n* = 30) under stress and after recovery, respectively. Within each series, different capital or lowercase letters indicate significant differences among treatments (*P* < 0.05; one-way ANOVA followed by LSD)

Physiological measurements were taken on the two water-stress levels and after re-watering. In consonance with the water stress severity, midday water potential decreased significantly compared to control plants (*P* < 0.05) reaching − 1.01 ± 0.03 MPa in mild-WS and − 2.06 ± 0.09 MPa in severe-WS (Fig. 3a). Consistently, net CO_2_ assimilation rates (*A*_N_) and stomatal conductance (*g*_s_) showed a marked and significant decrease in both stress levels (*P* < 0.05; Fig. 3b, c), *A*_N_ being reduced in more than ~ 70% and ~ 90%, in mild-WS and severe-WS, respectively, while *g*_s_ was almost completely supressed in both treatments. Leaf relative water content (RWC) decreased only significantly (*P* < 0.05) in the severe-WS treatment showing values of 87.5 ± 0.85% whereas in control and mild-WS, values were ~ 94%.

At the photochemical level, dark-adapted photochemical efficiency of photosystem II (PSII; *F*_v_*/F*_m_) was not significantly affected by water stress and mean values were close to 0.82 in all treatments (Table 1), indicating that water stress levels did not entail chronic photo-inhibition. The relative quantum yield of PSII photochemistry (ΦPSII) was not affected in the mild-WS treatment but was significantly reduced in the severe-WS (Table 1). Water stress treatments did not have an effect on the fraction of PSII centres in the open state (*q*L [47];) while the reverse was true for the maximum photochemical efficiency of the open reaction centres of PSII (*F*_v_’/*F*_m_′), which was significantly reduced as water stress became more severe (Table 1). These changes in *F*_v_’/*F*_m_′ were accompanied by a concomitant increase in other non-photochemical quenching related parameters (NPQ and *q*N; Table 1).

**Table 1:** Maximal photochemical efficiency of PSII (*F*_v_/*F*_m_), relative quantum yield of PSII photochemistry (ϕPSII), maximum photochemical efficiency of the open reaction centres of PSII (*F*_v_’/*F*_m_′), fraction of PSII centres in open state (*q*L), non-photochemical fluorescence quenching (NPQ) and coefficient of non-photochemical fluorescence quenching (*q*N) in control non-stressed plants, water stressed plants (mild-WS and severe-WS) and drought-primed plants (recovery mild-WS and severe-WS)

	Control			Mild-WS			Severe-WS			Recovery Mild-WS			Recovery Severe-WS		
*F*_*v*_ */F*_*m*_	0.821	±	0.00	0.820	±	0.00	0.817	±	0.00	0.825	±	0.00	0.825	±	0.00
*ϕ PSII*	0.566	±	0.01^a^	0.556	±	0.01^a^	0.459	±	0.02^b^	0.562	±	0.01^a^	0.547	±	0.01^a^
*F*_*v*_ *’/F*_*m*_ *′*	0.696	±	0.01^a^	0.661	±	0.01^b^	0.597	±	0.01^c^	0.703	±	0.01^a^	0.698	±	0.01^a^
*qL*	0.587	±	0.03^ab^	0.649	±	0.03^a^	0.584	±	0.03^ab^	0.550	±	0.02^b^	0.533	±	0.03^b^
*NPQ*	0.510	±	0.03^c^	0.785	±	0.05^b^	1.224	±	0.09^a^	0.581	±	0.05^c^	0.599	±	0.04^c^
*qN*	0.413	±	0.02^c^	0.534	±	0.02^b^	0.655	±	0.02^a^	0.439	±	0.02^c^	0.455	±	0.02^c^

Each value is the mean ± SE (controls *n* = 36, treatments *n* = 38). Different letters indicate significant differences among treatments within rows (*P* < 0.05)

Relative chlorophyll content (SPAD index) and leaf mass area (LMA) did not differ significantly between control and water stressed plants and no symptoms of leaf chlorosis were observed in any of the water stress treatments. Average SPAD values in all treatments were 59.4 ± 0.1 and LMA ranged from 76.8 g m^− 2^ to 83.4 g m^− 2^.

Within one week after re-watering and prior to inoculation with *R. necatrix*, all physiological parameters of stressed plants recovered similar values to those of control plants (Table 1 and Fig. 3). Hereinafter, these water-stressed-recovered plants will be referred as ‘*primed plants’*.

### Molecular response of avocado ‘Dusa’ rootstocks to mild and severe water stress and recovery after re-watering

The expression of thirteen defense-related genes on roots of ‘Dusa’ avocado rootstock subjected to mild-WS and severe-WS and one week after re-watering, was analysed by performing a real time quantitative qPCR (qRT-PCRs). This selection included induced genes indicated in previous studies with ‘BG83’ (tolerant to *R. necatrix*) and ‘Dusa’ (tolerant to *P. cinnamomi*) avocado rootstocks after infection with the soilborne pathogens *R. necatrix* [43] and *P. cinnamomi*, respectively [19, 48–50]. In addition to their implication in pathogen defense, some of the selected genes are also involved in salt, oxidative, osmotic and water stress responses (Table 2).

**Table 2:** qRT-PCR expression data of selected contigs from non-inoculated ‘Dusa’ roots subjected to two different level of water stress (mild-WS and severe-SW) and after their recovery (primed plants)

Citation of pathosystems					Mild-WS		Severe-WS	
BG83/*R. necatrix*	Dusa/*P. cinnamomi*	Contig/ GenBank ID	Annotation	Additional feature	Stress	Recovery	Stress	Recovery
[43]	[19]	Pa_Contig02817	Basic 7 s globulin-like	Salt and osmotic stress [53]	**−1.80** ± **0.37**	2.40 ± 0.60	7.11 ± 3.54	4.27 ± 2.63
[43]		Pa_Contig00582	BTB/POZ and TAZ domain-containing prot. 1-like	Salt stress [54]	**−10.16** ± **1.81**	**−1.93** ± **0.32**	**−12.76** ± **0.48**	**−2.52 ± 0.65**
	[19, 48, 49]	Pa_Contig00535	Endochitinase	Salt stress response [55]	1.28 ± 0.10	**2.35** ± **0.38**	1.29 ± 0.16	3.29 ± 1.15
	[48, 49]	Pa_Contig00778	Glutathione s-transferase	Salt, water and oxidative stress [56]	**2.17 ± 0.25**	−2.03 ± 0.83	**2.75 ± 0.25**	3.16 ± 0.50
	[48, 49, 51]	Pa_Contig04910	Metallothionein-like prot.	Oxidative and water stress [57, 58]	**2.79** ± **0.27**	**1.92 ± 0.17**	**1.48** ± **0.06**	2.69 ± 0.70
[43]	[19]	Pa_Contig02540	Miraculin	Water stress [59]	**2.38 ± 0.35**	1.21 ± 0.10	**3.25** ± **0.48**	**1.92** ± **0.00**
[43]		Pa_Contig00313	NAC domain-containing prot. 72	Salt and water stress [60–62]	**7.32** ± **1.38**	**177.00 ± 1.06**	**17.88** ± **1.43**	3.27 ± 1.49
	[52]	KR056089	NPR1	Salt and osmotic stress [63]	**−1.33** ± **0.19**	**1.21** ± **0.01**	**−2.04** ± **0.15**	**−2.71 ± 0.34**
[43]	[49]	Pa_Contig07140	PR4	Salt and water stress [64]	**−1.91 ± 0.17**	**6.19** ± **0.44**	1.31 ± 0.15	2.80 ± 0.86
	[48, 49, 51]	Pa_Contig01450	PR5	Salt and osmotic stress [65]	**−1.51 ± 0.18**	**1.72** ± **0.12**	−1.76 ± 0.30	3.06 ± 1.51
	[51]	Pa_Contig03407	PR10 (PsemI)	Salt and water stress [66]	**−2.22** ± **0.52**	**−1.99 ± 0.15**	**−1.78 ± 0.44**	1.40 ± 0.16
[43]	[19]	Pa_Contig05213	Protease inhibitor-like	Oxidative stress [45]	**1.72 ± 0.15**	**2.84** ± **0.56**	**4.92** ± **0.61**	**4.28 ± 0.33**
[43]	[51]	Pa_Contig01245	Universal stress prot.	Oxidative and water stress [67]	**1.23 ± 0.06**	**2.41 ± 0.14**	**1.63 ± 0.09**	**2.07 ± 0.19**

The data are displayed as fold changes (FC) calculated by comparing treatments with non-stressed control plants. The expression data are the mean of three biological replicates with three technical replicates each. The numbers in bold indicate statistically significant results (t-test, *P* < 0.05)

**Fig. 4:**
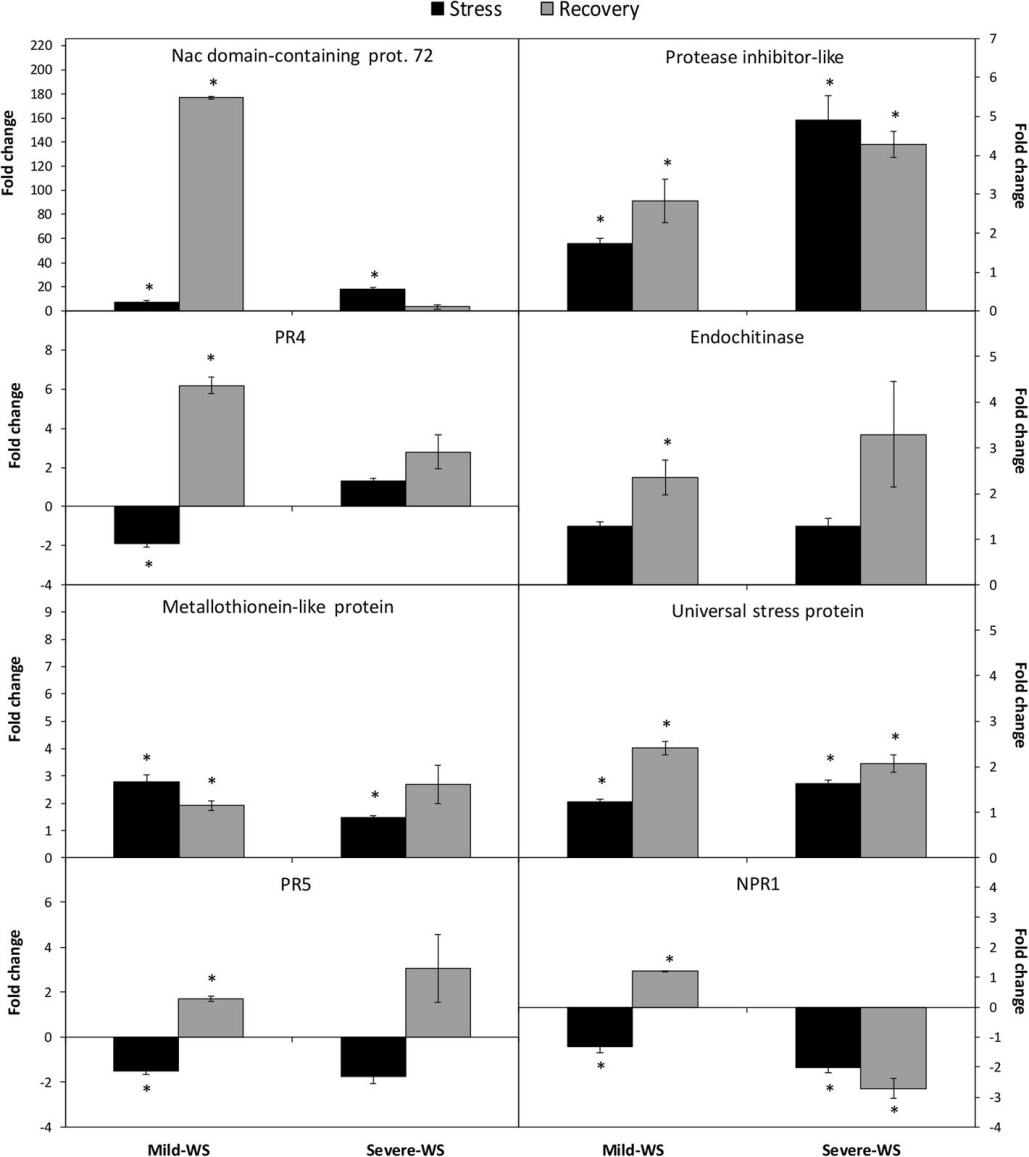
Gene expression analysis by qRT-PCR assay of eight selected genes in ‘Dusa’ plants subjected to two levels of water stress (WS), mild-WS and severe-WS and recovery after re-watering. Data are displayed as fold change (FC) calculated by comparing treatments with non-stressed control plants. The expression data are the mean (±SE, *n* = 9) of three biological replicates with three technical replicates each. Asterisk indicate statistical differences to control plants (Student’s t-test, *P* < 0.05)

Five primers were taken from literature and eight were developed in this research (Additional file 1: Table S1). The actin gene was used as an endogenous constitutive gene to normalize the expression results, and negative controls were used to confirm the absence of contamination. The relative quantification for the expression of the selected genes by the ΔΔCt method is shown in Table 2. Water deprivation on avocado roots caused a significant repression of 6 and 3 genes in roots subjected to mild-WS and severe-WS, respectively (Table 2), with gene Contig00582, encoding the BTB/POZ and TAZ domain-containing protein 1-like, showing the highest repression in both treatments. In contrast, transcript levels of 6 genes (protease inhibitor-like, glutathione s-transferase, metallothionein like protein, NAC domain-containing protein 72, universal stress protein and miraculin) were significantly induced under both levels of water stress (Table 2 and Fig. 4).

Different gene expression patterns were detected in avocado roots of primed plants, in which the number of significantly repressed genes was reduced to two in both, mild-WS and severe-WS primed plants. A higher number of significantly overexpressed genes was observed in roots of mild-WS primed plants, being induced eight genes among which, four were repressed under water stress (NPR1, PR4, PR5, endochitinase). The highest induction level was found for the NAC domain containing protein 72, reaching fold change (FC) value of 177 in qRT-PCR experiments. Only three of the study genes (protease inhibitor-like, universal stress protein and miraculin) were significantly induced in roots of severe-WS primed plants.

### Pathogenicity test on water stress primed ‘Dusa’ avocado rootstocks

**Fig. 5:**
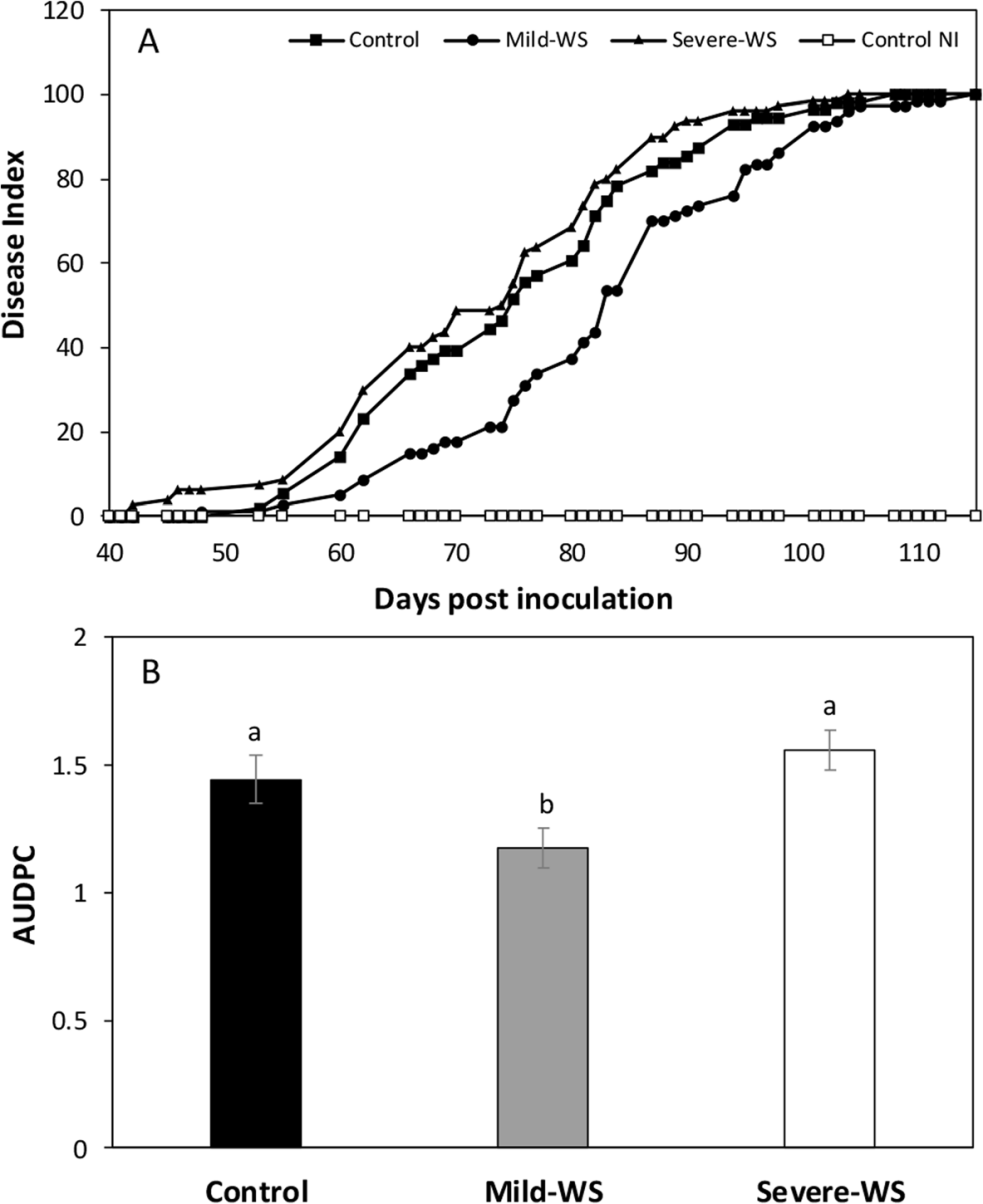
Disease index calculated by evaluating aerial symptoms in control, mild-WS and severe-WS primed ‘Dusa’ plants infected with *R. necatrix* (**a**), and mean values (±SE, controls *n* = 18 and treatments *n* = 20) of area under disease progress curve (AUDPC) for each treatment (**b**). Different letters indicate significant differences among groups (*P* < 0.05; one-way ANOVA followed by LSD)

In order to test whether priming with mild-WS and severe-WS could be used to induce tolerance to *R. necatrix* in avocado ‘Dusa’ rootstock, primed avocado plants were inoculated with wheat grains infected with *R. necatrix*. Disease progression was slightly faster in severe-WS primed plants than in non-primed control plants. Thus, visible aboveground WRR symptoms appeared 42 and 53 days post-inoculation, respectively. After 60 days post-inoculation, 50% of the non-primed control plants and severe-WS primed plants showed visible aerial symptoms (Fig. 5a).

Priming plants with mild-WS showed improved tolerance to WRR as indicated by a significant reduction in the area under disease progress curve (AUDPC) values (*P* < 0.05) (Fig. 5b). Although visible wilting symptoms in some leaves appeared 48 days post inoculation, 50% of the plants displayed aboveground WRR symptoms after 75 days post-inoculation (30 days after the first visible symptoms). After three and a half months post inoculation all non-primed control and severe-WS primed plants were at stage 5 (dead), while some mild-WS primed plants remained at stage 3.

## Discussion

Plants have evolved diverse strategies to cope with different environmental stresses, but many studies have shown that most plant responses to biotic and abiotic stress rely on an assortment of common physiological and molecular mechanisms [18, 19]. Particularly, it has been reported that avocado ‘Dusa’ rootstock response to *R. necatrix* infection involves the impairment of water relations and photosynthesis [68–70] as well as the induction of genes related to water stress and pathogen defense responses [43]. These findings are in agreement with results of the present study on the response of ‘Dusa’ avocado to water stress. This response was dependent upon water stress intensity, since mild-WS and severe-WS treatments affected leaf water status differentially (i.e. decreased values of leaf water potential and RWC) as well as photosynthetic performance, shown by the enhancement of photoprotective mechanisms (i.e. NPQ and *q*N values) and the decrease in gas exchange parameters (i.e. *A*_N_ and *g*_s_). These physiological changes are consistent to those previously described in response to mild and severe water stress in other woody plants [71, 72] and in avocado trees [73, 74]. ‘Dusa’ rootstock response to either *R. necatrix* infection or water stress treatments displayed water potential and *g*_s_ values that dropped below − 1.0 MPa and 0.05 mol m^− 2^ s^− 1^, respectively, suggesting an oxidative burst in photosynthetic tissues [75, 76]. This agrees with the higher NPQ and *q*N values [77, 78] and with a potential vulnerability to cavitation that could limit water flow from roots towards the upperparts of the trees, especially in severe-WS [79, 80]. In the *R. necatrix*/avocado interaction, this limitation of water flow is consistent with the profuse invasion of root vascular system during pathogen root colonization [70, 81].

Molecular responses at the root level showed the up-regulation of six out of the thirteen tested genes under both water stress treatments (Table 2). These genes, besides being involved in the avocado response to soilborne pathogens (*P. cinnamomi* and *R. necatrix*), are also induced in the response of other horticultural and woody species (i.e. *Citrus* spp., *Malus domestica*, *Populus trichocarpa*) to water deficit [45, 82–86]. It is remarkable the increased overexpression of NAC transcription factor accordingly to the intensity of the water stress level, which could be supporting a major accumulation of ROS species under severe-WS since, among other functions, this gene has been associated with the up-regulation of ROS-scavenging genes under abiotic stresses [61]. On the other hand, mild-WS repressed seven out of the thirteen genes, three of which remained down-regulated in the severe-WS (Table 2). NPR1 and PR5 repression is in consonance with the ABA biosynthesis and signalling induced under water stress [87], known to exert an antagonistic effect on the salicylic acid (SA) pathway [88] in which NPR1 functions as a master regulator inducing the expression of pathogenesis related proteins (PR) such as PR5 [89, 90], which are potentially involved in the maintenance of osmotic adjustment in cells [65].

The results stated above indicate that pathways involved in the avocado response to gradually imposed water stress lead to the induction of genes expressed in incompatible interactions against fungal pathogens [43, 90]. In this regard, co-occurrence of water stress and soilborne pathogens could have a positive effect in achieving tolerance against the pathogen (i.e. cross-tolerance, [20, 21]) or a negative additive effect, making plants more susceptible [16, 91–94]. Additional studies on avocado are required to clarify this point.

Previous studies have suggested the use of ‘priming’ [10–12] with drought stress to achieve tolerance to forthcoming diseases [16]. This acquired tolerance is based on sustained changes on the basal levels of cellular and molecular defense in primed plants after cessation of stimuli compared to non-primed ones [11, 12, 16]. In the present study, water status and photosynthetic performance was completely restored in drought-primed plants one week after re-watering regardless of the pre-drought intensity. This fast recovery suggests that impairment of whole plant transpirational flow and photosynthesis did not lead to irreversible changes on avocado and can be indicative of some degree of drought adaptation [78].

However, at the root level, re-watering induced the upregulation of defense related genes, suggesting a ‘primed state’ of the previously water stressed avocado plants. Gene overexpression, which could be associated with crosstalk between the different signaling pathways underlying plant tolerance/resistance to biotic and abiotic stress such as the abscisic (ABA), jasmonic (JA) and salicylic (SA) acids [95–98], was more remarkable in mild-WS compared to severe-WS. Particularly, the induction of NPR1 transcription factor in mild-WS primed plants suggests the activation of salicylic acid-mediated defense responses [52, 89, 90] and the deactivation of ABA-related responses after water stress [99]. In addition, this ‘primed state’ is accompanied by the significant accumulation of PR proteins (i.e. PR4 and PR5) which have been correlated with the development of systemic acquired resistance [48] and are considered the most promising candidates for developing multiple stress tolerance [89]. It is also remarkable that the expression of genes related with fungal cell wall degradation, such as endochitinase, was only up-regulated in plants recovered after mild-WS. Genes encoding metallothionein, universal stress protein, protease inhibitor and NAC domain containing protein 72 remained overexpressed in mild-WS primed plants. These genes are involved in the general plant response to stress [51, 58, 60, 62, 64, 67, 82, 100–103], playing the last two a fundamental role in avocado defense to *R. necatrix* [43]. It should be highlighted the marked overexpression of the gene encoding the NAC domain containing protein 72 (24 fold over mild-WS) in roots recovered from mild-WS compared to severe-WS, suggesting a higher promotion of root development [104, 105], although further studies are necessary to clarify its importance on the water stress recovery response.

The performed pathogenicity test shed light on whether this water-stress induced ‘primed state’ was effective for enhancing avocado tolerance to this necrotrophic pathogen. In this sense, the disease progression delay, observed in mild-WS primed plants in comparison with control and severe-WS primed plants, suggests an enhancement of plant ability to cope with *R. necatrix* infection after priming with mild water stress. This ability could be attributable to differential expression of key genes involved in the tolerance of avocado to soilborne pathogens such as NPR1 and NAC domain containing protein 72, as well as with a lower energy investment for overcoming a moderate water stress compared with severely stressed plants [72]. Moreover, although all of the overexpressed genes in mild-WS primed plants are involved in plant defense against fungi, not all have been described to be related with avocado tolerance to *R. necatrix* (i.e. NPR1, PR4, PR5 and endochitinase). However, their enhanced expression after drought-priming (i.e. abiotic factor) could also represent a benefit for avocado plants to overcome forthcoming fungal infection (i.e. biotic stressor).

## Conclusions

In conclusion, this is the first study reporting the effectiveness of ‘*cross-factor priming’* on the susceptible avocado rootstock ‘Dusa’ for increasing its tolerance to white root rot disease. Mild-WS induced a primed state in the WRR susceptible avocado rootstock ‘Dusa’ by overexpressing fungal defense related genes, revealing that plant responses against biotic and abiotic stress rely on common mechanisms. Although future experiments must be carried out on grafted plants, results presented here indicate the possibility of using moderate water stress as an approach to reduce *R. necatrix* impact on avocado orchards infected with the pathogen. These results reinforce the use of deficit irrigation strategies for disease management and water savings in cropping areas with limited water resources [74].

## Methods

### Plant material and experimental design

In order to test if water stress can be used as a priming factor for improving avocado tolerance to *R. necatrix*, a ‘*cross-factor priming*’ experiment was carried out in 2017 at the Institute of Agricultural Research and Training (IFAPA) (Málaga, south-eastern Spain, 36° 40′ 25″ N, 04° 30′ 11″ W, elevation of 32 m below sea level). One hundred and twelve 2-year old clonal ‘Dusa’ plants (Westfalia Estate, South Africa) propagated by Brokaw nursery (Brokaw España S.L.) using a modified Frohlich method [106], were grown in 16 L pots containing a sterilised mixture of organic substrate and sand supplemented with a slow-release fertiliser (Basacote Plus 6 M, Compo Expert GmbH).

‘Dusa’ plants were kept in a greenhouse under day light illumination and semi-controlled conditions of air temperature (T) and relative humidity (RH). Photosynthetic photon flux density (PPFD), T and RH conditions inside the greenhouse were continuously registered by a quantum sensor (Apogee SQ-110, USA) and by a T/RH U23–001 HOBO® Pro v2 logger (Onset Computer Corporation, USA). Maximal midday values of PPFD varied between 440 and 1012 μmol m^− 2^ s^− 1^, and daily T was allowed to fluctuate according to external weather conditions, but its variation range inside the greenhouse was maintained between 20 ± 10 °C by an automatic cooling system and heating when necessary. The RH values inside the greenhouse were always over 40%.

The experimental design is depicted in Fig. 1. At the beginning of the experiment (t_0_), plant physiological status was tested non-destructively by measuring chlorophyll fluorescence at predawn. Plants were randomly distributed in rows into two sets of 56 plants to conduct two trials. For each trial, 18 plants were randomly assigned to a control group, in which soil moisture was maintained at field capacity (Fc) throughout the experimentation, and two sets of 19 plants were subjected to controlled substrate drying-up until they reached 50% of Fc (i.e. mild water stress, mild-WS) and 25% of Fc (i.e. severe water stress, severe-WS), respectively. Once these soil water content levels were attained (after ~ 16–17 days; t_1_), full irrigation was restored in all plants and drought recovery response was assessed one week after re-watering (i.e. after ~ 23–24 days; t_2_). Hereinafter, the term ‘*primed plants’* refers to plants subjected to each of the water stress levels followed by a recovery period. The pathogenicity test with *R. necatrix* was performed at t_2_ as described below.

Soil moisture was monitored in all plants with a wet sensor (HH2 Moisture meter, Delta-T Devices. Cambridge, England), previously calibrated for the substrate, which also allowed adjustment of volumetric soil moisture (v/v) for each water treatment (mild-WS and severe-WS) in relation to the soil water holding at field capacity (Fc~ 0.4 v/v). Once per week plants were fertilised with an NPK solution (Kristalon Blue 17–6–18, Yara, UK) supplemented with iron chelate (Sequestrene®, Syngenta, Spain).

Throughout the experiment, physiological measurements and root samplings were carried out at t_1_ and t_2_. On each trial, 15 plants per treatment were measured at each sampling point. Roots were sampled from 9 plants per treatment not used for the pathogenicity test.

### Physiological measurements

Midday (12:00–14:00 am) leaf water potential was measured at t_1_ (when mild-WS and severe-WS plants reached 50 and 25% of Fc) and at t_2_ (one week after re-watering) using a Schölander pressure chamber (model 3005; Soil Moisture Equipment Corporation, Santa Barbara, CA, USA). On each trial, 15 plants per treatment were measured at each sampling point. Measurements were done in one mature fully developed leaf per plant close to the main stem. After cutting, leaves were immediately placed in the chamber following the recommendations made by Hsiao [107].

Relative leaf water content (RWC), the specific leaf mass area (LMA) and relative chlorophyll content (SPAD index) were measured only at t_1_ in the same plants as for leaf water potential determinations. For RWC determinations, leaf discs (2 cm^2^) were sampled at midday, weighed to obtain fresh weight (F_W_) and immediately imbibed on distilled water for 24 h at 5 °C in darkness for obtaining turgid weight (T_W_). Afterwards, samples were oven dried at 80 °C for 48 h to get dry weight (D_W_). RWC was calculated as follows:

$$ \mathrm{RWC}\ \left(\%\right)=\left[\left({\mathrm{F}}_{\mathrm{W}}-{\mathrm{D}}_{\mathrm{W}}\right)/\left({\mathrm{T}}_{\mathrm{W}}-{\mathrm{D}}_{\mathrm{W}}\right)\right]\times \kern0.37em 100 $$

The specific leaf mass area (LMA) was calculated as the ratio between disc dry weight and disc area (g cm^− 2^).

The SPAD index was non-destructively measured at midday on one leaf per plant using a hand-held SPAD 502 m (Minolta, Osaka, Japan). This index provides an estimation of leaf chlorophyll content consistent with leaf greenness [108]. For each plant, averaged SPAD values were calculated from three readings per leaf.

In vivo chlorophyll *a* fluorescence signals were measured with a portable fluorometer PAM-2100 (Heinz Walz, Effeltrich, Germany) at predawn (at t_0_) and midday (at t_1_ and t_2_) in one leaf per plant. The so-called saturation pulse method was used to determine all fluorescence parameters [109]. Dark-adapted parameters (i.e. minimal fluorescence (*F*_0_), maximal fluorescence (*F*_m_) and maximal photochemical efficiency of PSII (*F*_v_*/F*_m_ *= [F*_m_ *− F*_0_*]/F*_m_) were determined at predawn (05:00–07:00 am). The steady-state fluorescence (*F*_t_), maximal fluorescence (*F*_m_′) and minimal fluorescence yield of a pre-illuminated sample (*F*_0_*’*) were assessed in light acclimated leaves (∼450 μmol quanta m^− 2^ s^− 1^). The relative quantum yield of PSII photochemistry (ΦPSII = [*F*_m_′ *− F*_t_]*/F*_m_′) [110], the fraction of PSII centres in open state (*q*L) [47] and the extent of “Stern-Volmer” non-photo-chemical fluorescence quenching (NPQ = [*F*_m_ − *F*_m_′]/[*F*_m_′]) [111] were calculated.

Leaf gas exchange was measured at midday (11:00–14:00 am) at t_1_ and t_2_ in one mature exposed leaf. Measurements were performed with an open portable photosynthesis system (model LI-6400, LI-COR, USA) equipped with a LED-light source (6400–02B), coupled to a sensor head/IRGA, and with a CO_2_ mixer (6400–01) to modify the incoming air’s CO_2_ concentrations. The operating flow rate was 500 mL min^− 1^ and CO_2_ partial pressure was 400 ppm. Saturating photosynthetic photon flux density (1000 μmol m^− 2^ s^− 1^) was chosen as the default condition. Leaf temperature was kept at ∼20 °C and relative humidity was adjusted to 50% (vapor pressure deficit ∼1.4 kPa). Net CO_2_ assimilation rates (*A*_N_) and stomatal conductance (*g*_s_) were estimated with the equations of Von Caemmerer and Farquhar [112].

### RNA extraction

Roots from 9 avocado plants from control, mild-WS and severe-WS were harvested at t_1_ and t_2_ in plants others than those used in the pathogenicity test. Three biological replicates were used for RNA extraction. Each replicate consisted in a bulk sample from three plants. RNA from ground root tissue was extracted using the CTAB extraction method [113], a simple and efficient method for isolating RNA from pine trees with slight modification. The chloroform:isoamyl alcohol step was repeated 3–5 times, depending on the stability of the interphase and colour of the sample. RNA quantity and quality were determined based on A260/280 and A260/230 wavelength ratios using a NanoDrop® ND-1000 (Nanodrop Technologies, Inc., Montchanin, USA) spectrophotometer. RNA integrity was confirmed by the appearance of ribosomal RNA bands and lack of degradation products after separation on a 2% agarose gel and Red Safe staining. DNase treatment of RNA was performed by the addition of 1 U RNase-free DNase (Thermo Scientific, Life Technologies Inc., Carlsbad, California, USA), 1 μL 10x reaction buffer with MgCl_2_, 1 μg RNA, 0.5 μL of RiboLock RNase Inhibitor (Thermo Scientific Inc., California, USA) and diethylpyrocarbonate-treated water to a final volume of 10 μL. The mixture was incubated at 37 °C for 45 min followed by the addition 1 μL of 50 mM EDTA and incubation at 65 °C for 10 min.

### Quantitative real-time PCR

Single stranded cDNA was synthesized using iScript Reverse Transcription Supermix (Bio-Rad Laboratories Inc., California, USA) according to manufacturer’s instructions. The cDNA was analysed for genomic DNA contamination by PCR using gene specific primers F3H-F (5′–TCTGATTTCGGAGATGACTCGC–3′) and F3H-R (5′–TGTAGACTTGGGCCACCTCTTT–3′), which flank an intron of the eflavone 3-hydroxylase (F3H) gene. PCR amplifications were carried out as previously described by Engelbrecht and van den Berg [48] using first-strand cDNA as the template.

The expression of thirteen avocado genes was investigated based on previous literature. The actin gene was used as endogenous control for normalization. Primer sequences for endogenous control gene and the thirteen avocado genes are presented in Additional file 1: Table S1. Primer pairs were chosen to generate fragments between 70 to 140 bp and were designed using Primer 3 software (http://bioinfo.ut.ee/primer3–0.4.0/, [114, 115]). Primer specificity was tested by first performing a conventional PCR and confirmed by the presence of a single melting curve during qRT-PCR. Serial dilutions (1:10, 1:20, 1:50, 1:200) were made from a pool of cDNA from each treatment and time-points, and calibration curves were performed for each gene. For qRT-PCR, the reaction mixture consisted of cDNA first-strand template, primers (500 nmol final concentration) and SYBR Green Master Mix (SsoAdvanced Universal SYBR Green Supermix, Bio-Rad) in a total volume of 20 μl. The PCR conditions were as follows: 30 s at 95 °C, followed by 40 cycles of 15 s at 95 °C and 30 s at 60 °C, 3 min at 72 °C, 1 min at 95 °C. The reactions were performed using an iQ5 real-time PCR detection system (Bio-Rad). Relative quantification of the expression levels for the target was analysed using the ΔΔCt method [116]. All reactions were done in triplicate.

### Pathogenicity test in avocado plants

Inoculum was produced on wheat seeds according to Sztejnberg and Madar [117]. Briefly, seeds were soaked for 12 h in 250 ml Erlenmeyer flasks filled with distilled water. The flasks, each containing 100 g of seeds, were subsequently autoclaved after excess water drained off. After sterilisation, four 0.5 cm diameter fungal discs of a 2-week-old culture of *R. necatrix* grown on potato dextrose agar (PDA) were placed aseptically in each flask and incubated at 24 °C in the dark for three weeks until wheat grains were homogeneously covered by *R. necatrix* mycelium. Seven days after re-watering (t_2_), ‘Dusa’ rootstocks from each treatment (control *n* = 9, mild-WS *n* = 10, severe-WS n = 10) on each of the two trials, were inoculated with 3.75 g of colonized wheat seeds per litter of substrate. To ensure the spread of the inoculum, it was placed at eight points scattered around the stem (~ 3.5 cm apart) and introduced at two depths (~ 5 cm and ~ 15 cm, respectively). Disease progression was evaluated by measuring the aerial symptoms of WRR according to a scale: 1 = healthy plant; 2 = mild wilting; 3 = wilting; 4 = desiccated; and 5 = death. The disease index (DI) for each treatment and the area under the disease progress curve (AUDPC) was calculated as previously described by Teixeira de Sousa [118] and Campbell and Madden [119], respectively.

### Statistical analysis

Data were analysed using the analytical software STATISTICA 7 (StatSoft, Inc., USA). Differences among treatments in physiological variables and AUDPC were evaluated by analysis of variance (ANOVA). On each sampling point, datasets obtained from the two trials were subjected to a two-way ANOVA, in which ‘trial’ and ‘treatment’ were the between-subjects factors. This analysis allowed to test whether the variability observed between the two trials was significantly different or not, and to what extent was it possible to merge datasets for performing a unique one-way ANOVA for each sampling point. Since no significant effect of ‘trial’ was observed in any of the variables analysed, data from the two trials were analysed jointly. Therefore, data depicted in the figures for each treatment are average values of the measurements taken in the two trials. Significant differences were considered at the 5% probability level unless otherwise stated. Prior to ANOVA, normality and homogeneity assumptions were tested by using the Kolmogorov–Smirnov and the Cochran’s C test, respectively. When significant differences were found, Fisher’s least significant difference (LSD) test was used to compare mean values. Statistical analysis of qRT-PCR data was carried out by Student’s t-test with Sigma Stat version 4.0 software (Systat Software GmbH).

## Additional file

Additional file 1: **Table S1.** Primers used in the qRT-PCR experiments. (DOC 67 kb)

## Data Availability

All data generated or analysed during this study are included in this published article [and its supplementary information files].

## Abbreviations

ABA: Abscisic acid
*A*_N_: net CO_2_ assimilation rates
AUDPC: area under disease progress curve
DI: disease index
D_W_: dry weight
Fc: Field capacity
FC: Fold change
*F*_v_/*F*_m_: Maximal photochemical efficiency of PSII
*F*_v_’/*F*_m_′: maximum photochemical efficiency of the open reaction centres of PSII
F_W_: Fresh weight
*g*_s_: stomatal conductance
JA: Jasmonic acid
LMA: Leaf mass area
NPQ: Non-photochemical quenching of fluorescence
NPR: Non-expressor of pathogenesis related
PDA: Potato dextrose agar
PPFD: Photosynthetic photon flux density
PR: Pathogenesis-related
PRR: Phytophthora root rot
PSII: Photosystem II
*q*L: Fraction of PSII centres in open state
*q*N: Coefficient of non-photochemical quenching
qRT-PCR: real time quantitative PCR
RH: Relative humidity
ROS: Reactive oxygen species
RWC: Relative water content
SA: Salicylic acid
T: Temperature
Tm: Primer melting temperature
T_W_: Turgid weight
WRR: White root rot
WS: Water stress
ϕPSII: Relative quantum yield of PSII photochemistry
