# Supplementary material for: Mild water stress-induced priming enhance tolerance to Rosellinia necatrix in susceptible avocado rootstocks
Source: BMC Plant Biol. 2019 Oct 29;19:458. doi: 10.1186/s12870-019-2016-3 (PMC6821026; doi:10.1186/s12870-019-2016-3)
Supplement: Supplementary file 1 — Table S1. Primers used in the qRT-PCR experiments. (DOC 67 kb) [file 12870_2019_2016_MOESM1_ESM.doc]

**Supplementary Table 1. qRT-PCR primer sequences used in this study.**

| **Description** | **Contig/ GenBank ID** | **Reference** | **Amplicon Tm (°C)** | **Amplicon size (bp)** | **Primer sequences (forward/reverse) [5**‘**-3**‘**]** |
| --- | --- | --- | --- | --- | --- |
| Actin | Pa_Contig00256 | [51] | 58 | 104 | CCAAGCAGCATGAAGATAAAGGT |
|  |  |  |  |  | CACATCTGTTGGAAGGTGCTC |
| Basic 7s globulin-like | Pa_Contig02817 | This research | 60 | 108 | TGCCACAGTTCCTCTTCTCC/ |
|  |  |  |  |  | GCGATGGGAGTCCAATTCTA |
| BTB/POZ and TAZ domain-containing prot. 1-like | Pa_Contig00582 | This research | 60 | 146 | TGGTCCTTGCAAAGTTCCTC/ |
|  |  |  |  |  | CTCCTCGCATTCTCTTGGAC |
| Endochitinase | Pa_Contig00535 | [48] | 57 | 83 | ATCACCAACATCATCAAC/ |
|  |  |  |  |  | CTCTTGTAGAAGCCAATG |
| Glutathione s-transferase | Pa_Contig00778 | This research | 60 | 140 | AAATCAGACGGTCATGGCGT/ |
|  |  |  |  |  | TCTTGTGATCCCCAGTTGCC |
| Metallothionein-like prot. | Pa_Contig04910 | [51] | 59 | 76 | AGTCTTCATCCCTAATACATATCCC/ |
|  |  |  |  |  | GTTTGTGCGTGTCTGGTTTC |
| Miraculin | Pa_Contig02540 | This research | 60 | 81 | TGAAGTGTCCAATGGCCTCC/ |
|  |  |  |  |  | TTGAAGTCCGTCGATGCCTG |
| NAC domain-containing prot. 72 | Pa_Contig00313 | This research | 60 | 115 | GACGACGTCCTCCTCGAATC/ |
|  |  |  |  |  | TTCCGAGGCTTTGATGGGTC |
| NPR1 | KR056089 | [52] | 56 | 119 | TGGCTTATCAGTGCTTGCTC/ |
|  |  |  |  |  | CCTCCTTATCCTCGTTGTATGC |
| PR4 | Pa_Contig07140 | This research | 60 | 136 | GTGCAACATGGGACGCTAAC/ |
|  |  |  |  |  | GCTCCTGTCCCTGTGTTTGT |
| PR5 | Pa_Contig01450 | [48] | 60 | 110 | CAAGAGTACGGACAGCCACC/ |
|  |  |  |  |  | TCCATGGGAATGTTGAAGCC |
| PR10 (PsemI) | Pa_Contig03407 | This research | 60 | 105 | TGAACGAACGGGTGGATGAG/ |
|  |  |  |  |  | TGTGATAAACGGCCGAGAGG |
| Protease inhibitor-like | Pa_Contig05213 | [43] | 60 | 93 | TACAGCCCAAATCGTCACGG/ |
|  |  |  |  |  | ACAATGCCTGCAGTCTCATCA |
| Universal stress prot. | Pa_Contig01245 | This research | 60 | 108 | TGGAGAGGGAGACTGGTGAG/ |
|  |  |  |  |  | TGCAGAAGTGAAGGATGGCC |
